# Supplementary material for: Targeting CXCL16 and STAT1 augments immune checkpoint blockade therapy in triple-negative breast cancer
Source: Nat Commun. 2023 Apr 13;14:2109. doi: 10.1038/s41467-023-37727-y (PMC10101955; doi:10.1038/s41467-023-37727-y)
Supplement: Supplementary file 1 — Supplementary Information [file 41467_2023_37727_MOESM1_ESM.pdf]

Targeting CXCL16 and STAT1 Augments Immune Checkpoint Blockade Therapy in Triple-Negative Breast Cancer

Bhavana Palakurthi[1, 2], Shaneann R. Fross[1,2], Ian H. Guldner[1, 2], Emilija Aleksandrovic[1, 2], Xiyu Liu[1, 2], Anna K. Martino[1], Qingfei Wang[1,2], Ryan A. Neff[1], Samantha M. Golomb[1,2], Cheryl Lewis[3], Yan Peng[3], Erin N. Howe[1, 2], and Siyuan Zhang[1, 2, 3, 4] *
Affiliations:
1. Department of Biological Sciences, College of Science, University of Notre Dame, Notre Dame, IN 46556, USA
2. Mike and Josie Harper Cancer Research Institute, University of Notre Dame, 1234 N. Notre Dame Avenue, South Bend, IN 46617, USA
3. Department of Pathology and Simmons Comprehensive Cancer Center, University of Texas Southwestern Medical Center, Dallas, TX 75235
4. Indiana University Melvin and Bren Simon Cancer Center, Indianapolis, IN 46202

* Correspondence to: Siyuan Zhang, M.D., Ph.D., Department of Pathology, University of Texas Southwestern Medical Center, Dallas, TX 75235
E-mail: Siyuan.Zhang@UTSouthwestern.edu; Telephone: 214-648-6537

This supplemental data file contains:
Supplemental Figures 1 - 6
Supplemental Tables 1- 2

Supplementary Figure 1

MCT increases T - myeloid cell spatial proximity

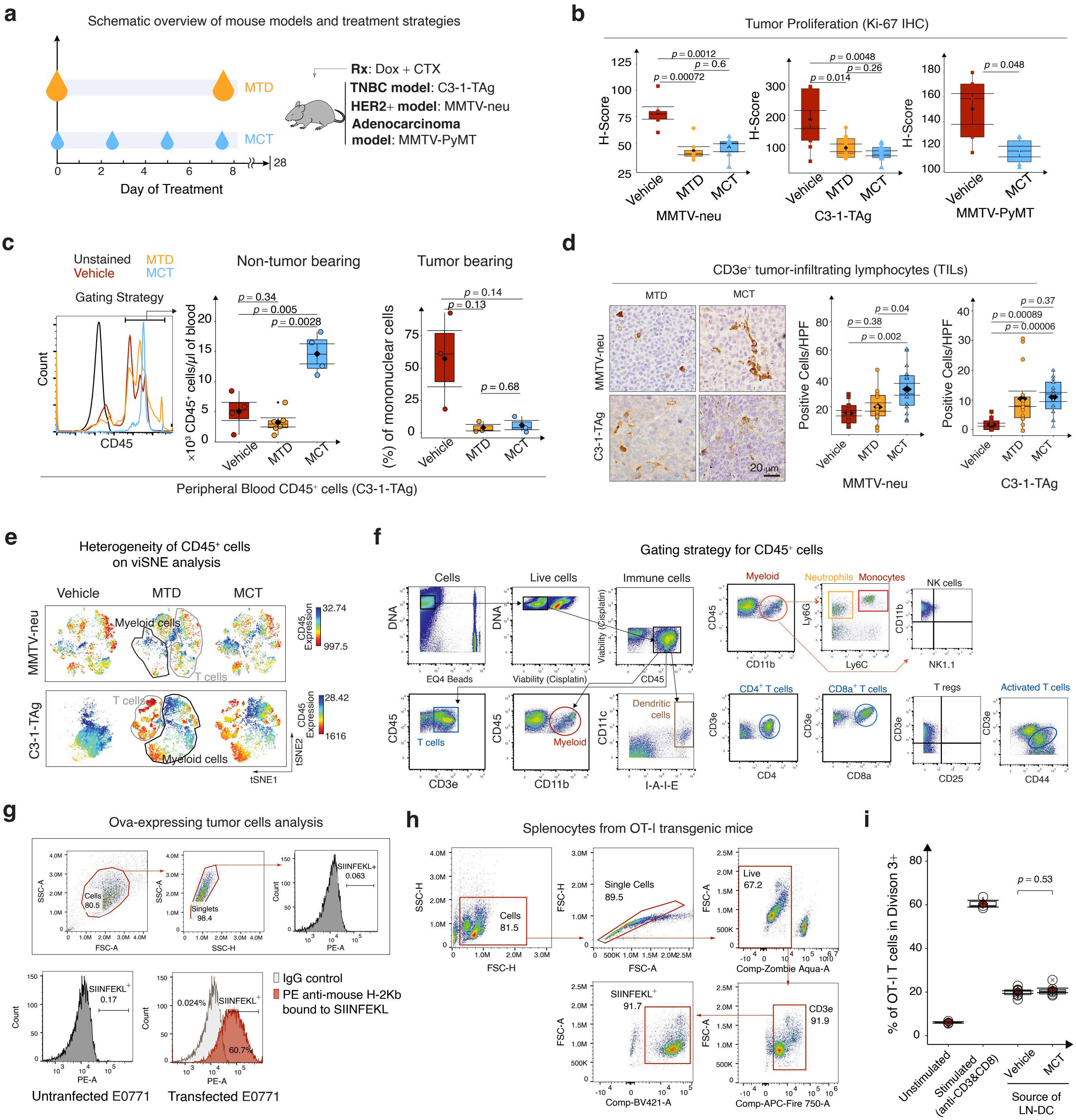

Figure S1. MCT increases T - myeloid cell spatial proximity. (a) Schematic overview shows the MTD and MCT treatment strategies. (b) H-Score quantification of mouse breast tumor tissues stained with Ki-67 in box plots (*n* = 6 to 9 slides per group) (c) Flow cytometry gating strategy to identify CD45$^+$ cells and quantification of CD45$^+$ cells in the peripheral blood of non-tumor and tumor bearing mice. (d) Representative IHC images (left) of MMTV-neu (top) and C3-1-TAg (bottom) stained with anti-CD3 and box plots (right) quantify CD3$^+$ cells per high power field (HPF) (*n* = 15 slides per group). (e) t-SNE plots show the tumor-associated immune cell distribution an their CD45 expression (*n* = 1 million cells per group including all the events). (f) CyTOF data biaxial plots show the gating strategy to identify different tumor-associated live immune cell populations. (g) Validation of ova expressing tumor cells. Upper panel: Gating strategy to identify SIINFEKL$^+$ E0771 tumor cells. Lower panel: Flow analysis of SIINFEKL expressing E0771 cells. (h) Biaxial plots show the gating strategy to identify the SINFEKL$^+$ viable unstimulated OT-I T cells isolated from OT-I transgenic mice. (i) CFSE T cell proliferation analysis of OT-I cells co-cultured with LN-DC: quantification of OT-I T cells that have undergone more than 3 divisions (*n* = 3 to 8 per group). (A-I) In box plots, error bar = Mean ± SEM, center = Means (Diamonds) and Medians (solid line), bottom and top boundaries of the box = 25 and 75th percentiles of the data, minima and maxima = lowest and highest data points, and two-sided T-test *p*-values.

Supplementary Figure 2

CITE-seq reveals co-existence of distinct TME myeloid cells

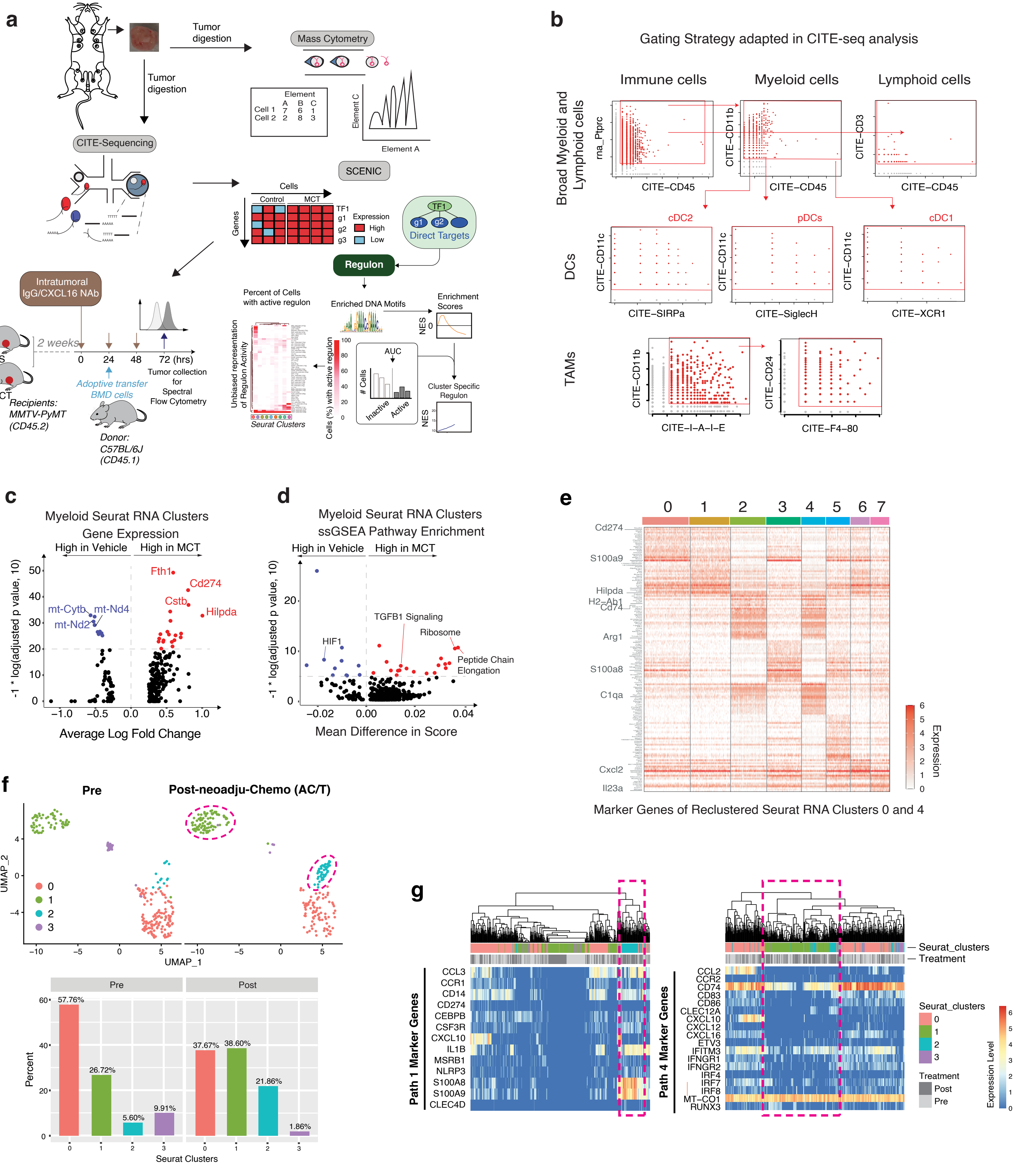

Figure S2. CITE-seq reveals distinct myeloid subsets coexist in MCT TME. (a) Schematic shows methodology. (b) Gating strategy adopted in CITE-seq analysis. (c-d) Volcano plots of DEGs and enriched genesets between MCT and Vehicle treated Seurat Myeloid clusters from Fig. 2c. (e) Heatmap shows top DEGs between clusters identified in Fig. 2G . (f) UMAP split by treatment groups shows different myeloid cell subsets. Bar plot shows the percentage distribution of Seruat clusters in UMAP. (g) Heatmaps show enrichment of Mouse Path 1 (left) and Path 4 (right) marker genes in Clusters 2 and 1 respectively.

Supplementary Figure 3

Transcriptional Profile of Tumor-Infiltrating Myeloid Cells

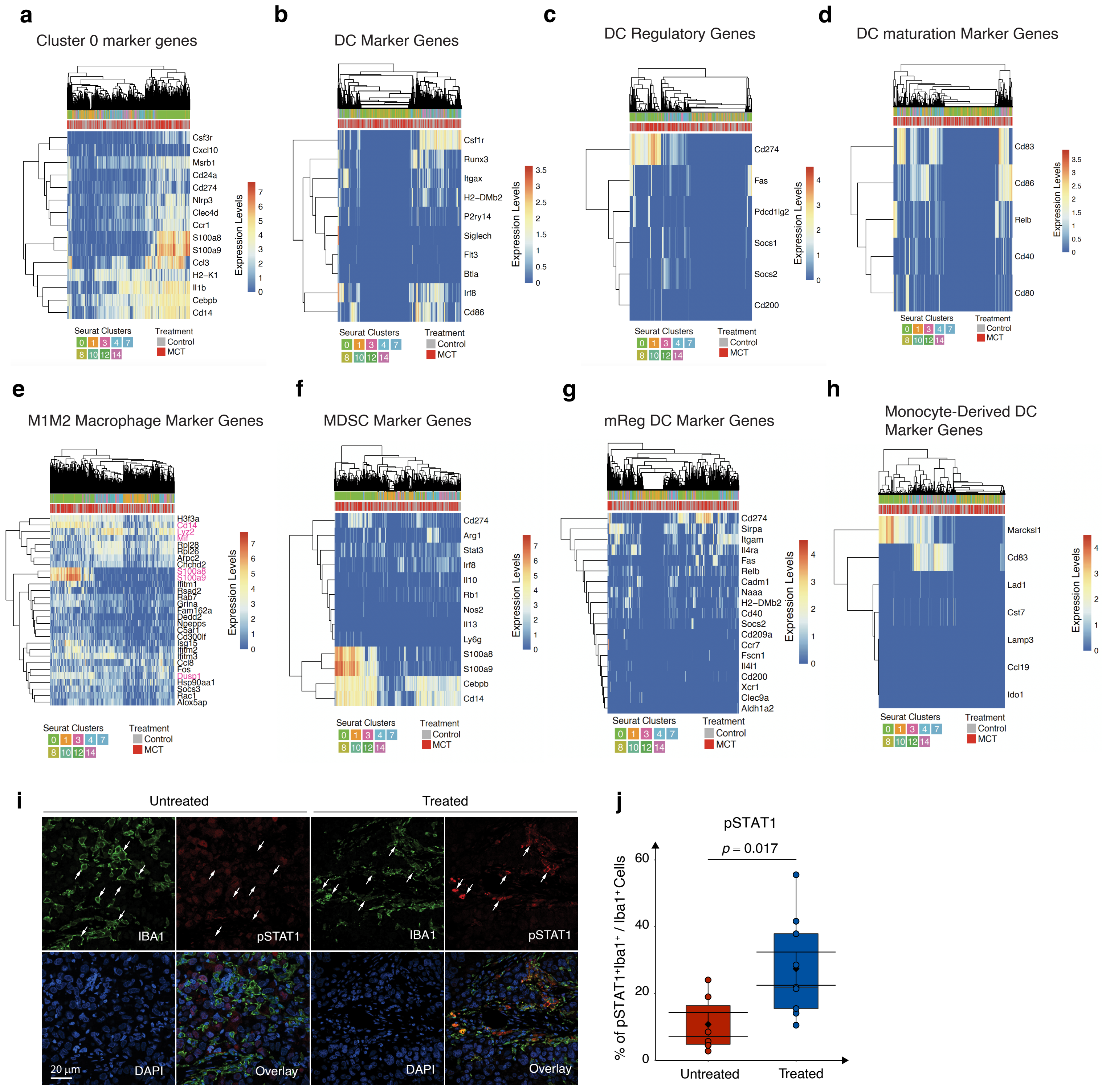

Figure S3. Transcriptional Profile of Tumor Infiltrating Myeloid Cells. (a-h) Transcriptional Profile of tumor-infiltrating myeloid cells after MCT treatment. Heatmaps show (a) Cluster 0 DEGs (b) DC (c) DC regulatory (d) DC maturation (e) M1/M2 macrophage (f) MDSC (g) mature regulatory (mreg) DC (h) Monocyte-Derived DC marker gene expression. (i-j) Representative images of Iba1, pStat1, and DAPI stain and their quantification of human breast cancer with and without ddACT chemotherapy. Data in boxplots represents quantification of random regions (n = 5 to 10) from four untreated and five treated patients. In box plots, error bar = Mean ± SEM, center = Means (Diamonds) and Medians (line), bottom and top boundaries of the box = 25 and 75th percentiles of the data, (whiskers) minima and maxima = lowest and highest data points, and two-sided T-test p-values. Also see associated Fig. S3 and Supplementary Table 7 and 8.See associated Supplementary Data Table 8.

Supplementary Figure 4

MCT-induced CXCL16 mediates intratumoral immune dynamics and ICB efficacy

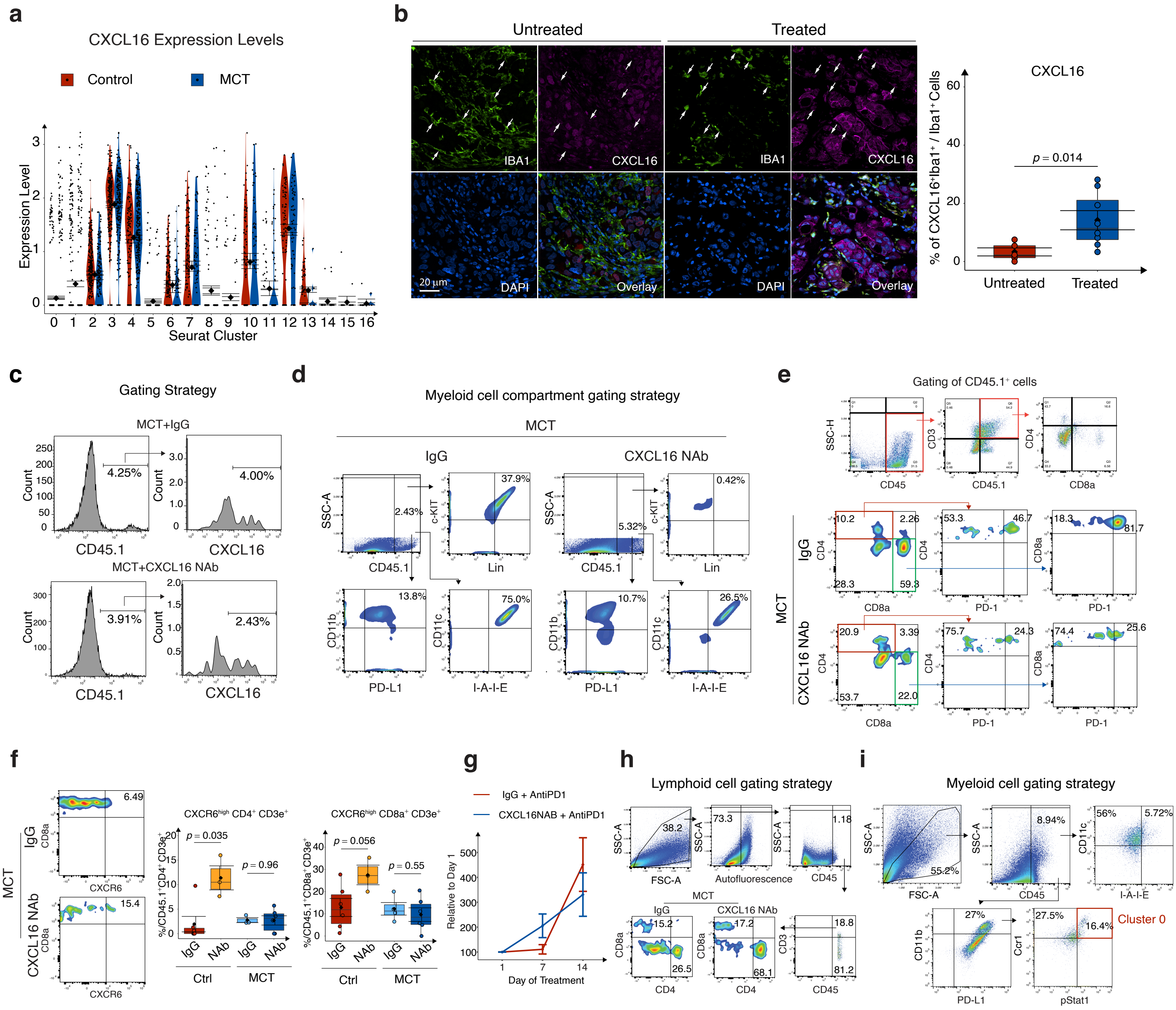

Figure S4. MCT-induced CXCL16 mediates intratumoral immune dynamics and ICB efficacy. (a) Violin plot compares expression of CXCL16 between Seurat clusters. $n = 3$ biological replicates per group. (b) Representative images of Iba1, CXCL16, and DAPI stain and their quantification of human breast cancer with and without ddACT chemotherapy. Data in boxplots represents quantification of random regions ($n = 3$ to 5) from four untreated and five treated patients. (c) Gating strategy to identify CXCL16⁺ CD45.1⁺ cells comparing between IgG and NAb groups of MCT-treated mice. (d) Gating strategy to identify c-kit⁺ Lin⁺, CD11b⁺PD-L1⁺, CD11c⁺ I-A-I-E⁺ cells (e) Gating strategy to identify CD4⁺, CD8a⁺, CD4⁺ PD-1⁺, CD8a⁺ PD-1⁺ (f) Gating of CXCR6⁺ CD4⁺ and CD8a⁺ T cells and quantification of CXCR6⁺ T cells (right). (g) Line plot for Fig. 4I shows the tumor volume growth over time. (h) Gating strategy to identify CD8 and CD4⁺ T cells infiltrating the tumors upon treatment with MCT and either IgG or CXCL16NAb. (i) Gating strategy to identify the Cluster 0 cells. In (a) violin plot, error bar = Mean ± SEM, center = Means (Diamonds), $n = 3$ biological replicates. (b-i) In box plots, $n = 3$ to 5 biological replicates, error bar = Mean ± SEM, center = Means (Diamonds) and Medians (Line), bottom and top boundaries of the box = 25 and 75th percentiles of the data, (whiskers) minima and maxima = lowest and highest data points, two-sided T-test p-values.

Supplementary Figure 5

Impact of Targeting STAT1 on PD-L1 expression and T cell activity

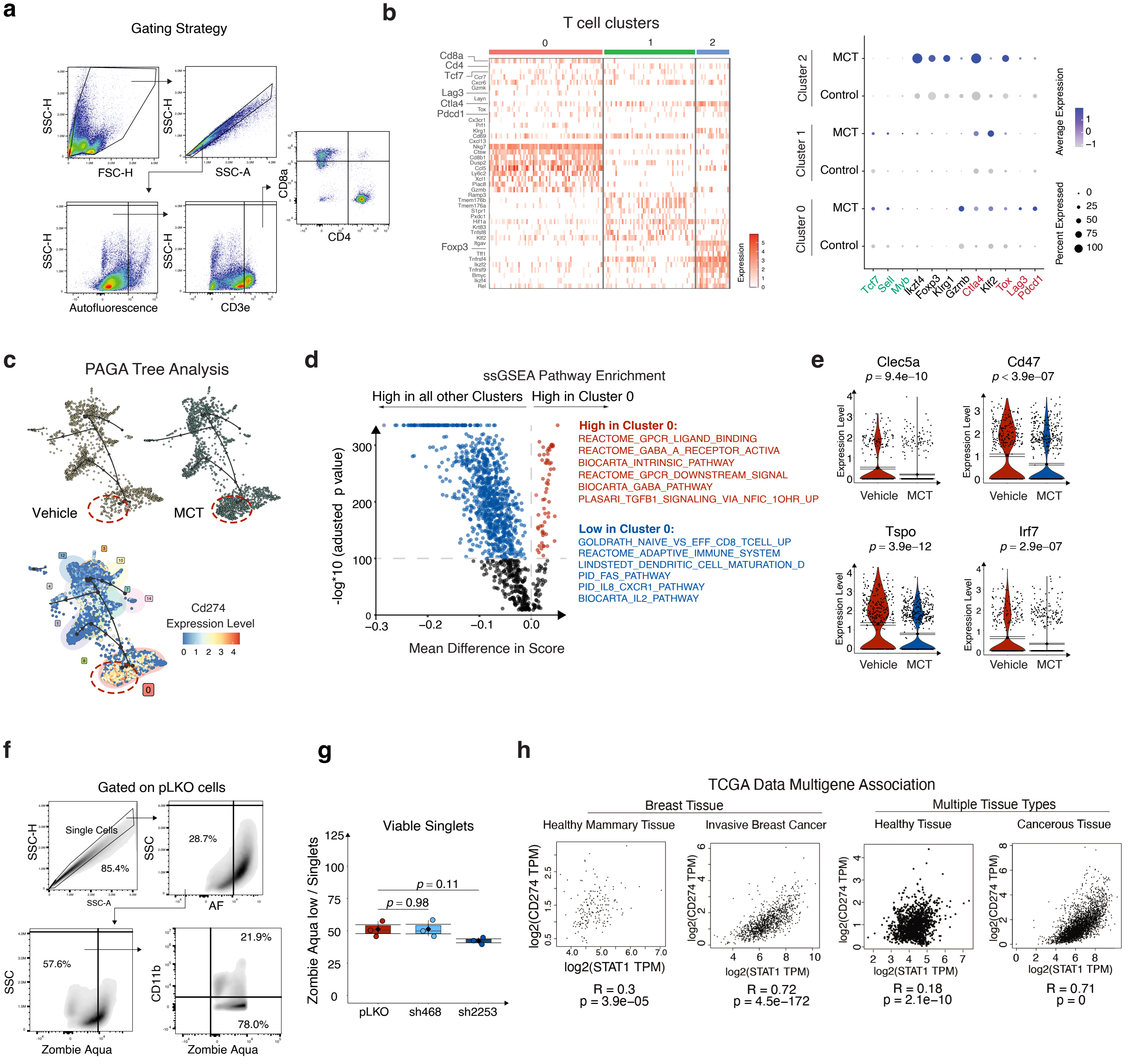

Supplementary Figure 5. STAT1 regulates PD-L1 expression in Cluster 0 cells and influences T cell activation status. (a) Gating strategy to identify CD8 and CD4 T cells in co-culture shown in Fig. 5a. (b) Heatmap (left) shows identity of different Cd3e ^{high} T cells. Dotplot (right) split by treatment group to show percentage of cells expressing various functional and differentially expressed genes. (c) PAGA trajectory trees (left, top) show Seurat Clusters (encircled are Cluster 0) split by treatment group and expression of Cd274 (left, bottom). (d) Volcano plot shows enriched DEGP from ssGSEA in Cluster 0 cells over other Seurat Clusters. (e) Violin plots show expression of Clec5a, Cd47, Tspo, and Irf7 genes by Cluster 0 cells (each dot is a cell and cells are pooled from $n = 3$ biological replicates, error bars = Mean ± SEM, diamonds = Mean). (f) Density plots show the gating strategy for viable CD11b^{+} cells in shRNA pLKO *in vitro* cultures. (g) Box plot shows the viable singlet percentage in diffferent shRNA constructs. In box plots, error bar = Mean ± SEM, center = Means (Diamonds) and Medians (Line), bottom and top boundaries of the box = 25 and 75th percentiles of the data, (whiskers) minima and maxima = lowest and highest data points, and two-sided T-test *p*-values. (h)Scatter plots show association between Stat1 and Cd274 across healthy mammary and invasive breast cancer tissue and multiple healthy tissues and their respective cancerous tissues. The plots were generated using Gepia web server and R is Spearman's correlation.

Supplementary Figure 6

Modulating STAT1 signaling enhances anti-PD-1 antibody mediated anti-tumor responses

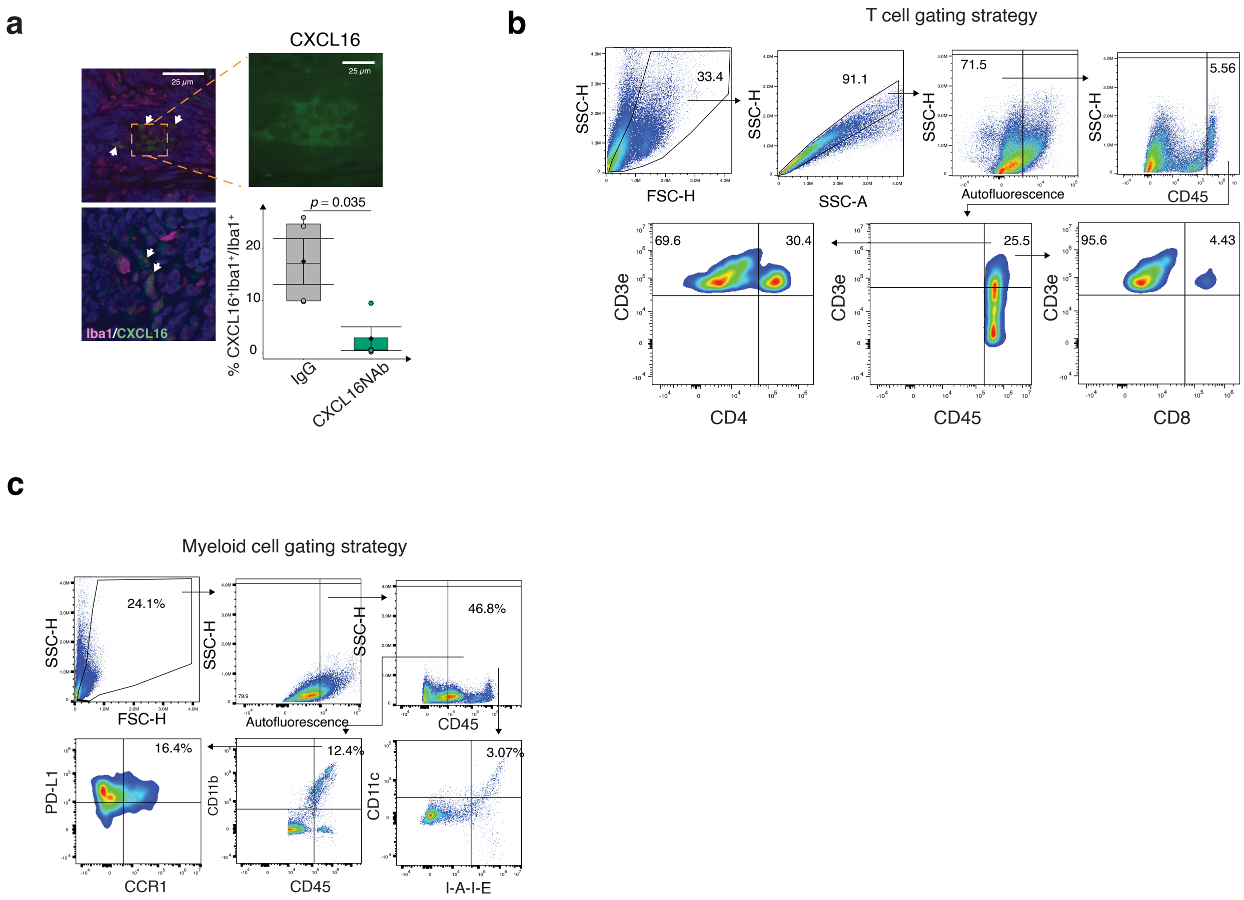

Figure S6. Modulating STAT1 signaling enhances anti-PD-1 antibody mediated anti-tumor responses. (a) Representative IF images show and box plots quantify the CXCL16⁺ myeloid cells upon treating with CXCL16 NAb (Regimen 2). (b) Flow plots show gating strategy to identify T cells. (c) Flow plots show gating strategy to identify myeloid cells. In box plot (A), $n = 3$ to 5 biological replicates, error bar = Mean ± SEM, center = Means (Diamonds) and Medians (line), bottom and top boundaries of the box = 25 and 75th percentiles of the data, minima and maxima = lowest and highest data points, and two-sided T-test *p*-values.

Supplementary Table 1: CyTOF antibody panel. List of CyTOF antibodies used, the motivation behind their usage, and their Fluidigm catalogue number.

Antibody	Motivation	Fluidigm #
CD45 - 089Y	General Immune markers	3089005B, 30-F11
CD45R - 144Nd	B cells	3144011B, RA3-6B2
Ly-6G - 141Pr	Neutrophils	3141008B, 1A8
Ly-6C - 162Dy	Monocytes	3162014B, HK1.4
CD11b - 148Nd	Myeloid Marker	3148003B, M1/70
NK1.1 - 165Ho	Natural Killer Cell Marker	3165018B, PK136
I-A/I-E - 209Bi	Major Histo Compatibility Complex 2	3209006B, M5/114.15.2
CD11c - 967 142Nd	Dendritic Cell	3142003B, N418
CD3e - 152Sm	General Lymphoid Marker	3152004B, 145-2C11
CD4 - 145Nd	T helper or T reg cell	3145002B, RM4-5
CD25 - 151Eu	T cell activation	3151007B, 3C7
CD44 - 150Nd	T cell activation	3150018B, IM7
CTLA-4 - 154Sm	Costimulatory Signaling	3154008B, UC10-4B9
CD8a - 168Er	Cytotoxic T cells	3168003B, 53-6.7
CD86 - 172Yb	Costimulatory Signaling	3172016B, GL1
CX3CR1 - 164Dy	General Myeloid Marker	3164023B, SA011F11
PD-1 - 159Tb	Co-inhibitory Signaling	3159024B, 29F.1A12
PD-L1 - 153Eu	Co-inhibitory Signaling	3153016B, 10F.9G2
CD117- ckit -166Er	Innate Lymphoid Cells	3166004B, 2B8

Supplementary Table 2: CITE-Sequencing antibody panel. List of CITE-Sequencing antibodies used, clones, oligonucleotide sequences, the motivation behind their usage, and their catalogue number.

Antibody	Clone	Olignucleotide Sequence	Motivation	Catalouge#
CCR2/CD192	SA203G11	AGTGCGATCTGCAAC	Peripheral macrophage	150625
CD117/c-kit	2B8	TGCATGTCATCGGTG	Innate lymphoid cells (ILCs)	105843
CD11b	M1/70	TGAAGGCTCATTTGT	General myeloid marker	101265
CD11c	N418	GTTATGGACGCTTGC	DCs	117355
CD172a/SIRP	P84	GATTCCCTTGTAGCA	Don’t eat me signal (SIRP)	144033
CD25	PC61	ACCATGAGACACAGT	T Cell activation	102055
CD3	17A2	GTATGTCCGCTCGAT	General T cell	100251
CD4	RM4-5	AACAAGACCCTTGAG	T helper or T reg	100569
CD44	IM7	TGGCTTCAGGTCCTA	DC activation marker	103045
CD45	30-F11	TGGCTATGGAGCAGA	General immune cell marker	103159
CD45R/B220	RA3-6B2	CCTACACCTCATAAT	B cells,pDCs	103263
CD86	GL-1	CTGGATTTGTGTATC	Costimulation	105047
CD8a	53-6.7	TACCCGTAATAGCGT	Cytotoxic T cells, pDCs	100773
CD90.1	OX-7	AGTATGGGATGCAAT	DC activation	202547
Cx3cr1	SA011F11	CACTCTCAGTCCTAT	DC chemokine receptor	149041
F4/80	BM8	TTAACTTCAGCCCGT	General macrophage	123153
I-A/I-E	M5/114.15.2	GGTCACCAGTATGAT	MHC II, high in cDCs	107653
Ly6C	HK1.4	AAGTCGTGAGGCATG	Monocyte subset	128047
Ly6G	1A8	ACATTGACGCAACTA	Neutrophils	127655
NK1.1	PK136	GTAACATTACTCGTC	NK cells	108755
PD-1	RMP1-30	GAAAGTCAAAGCACT	Co-inhibitory signaling	109123
PD-L1	MIH6	TCGATTCCACCAACT	Co-inhibitory signaling	153604
CD169/Siglec-1	3D6.112	ATTGACGACAGTCAT	Macrophage cell adhesion,self-tolerance	142425
Siglec-H	551	CCGCACCTACATTAG	pDCs	129615
XCR1	Zet	TCCATTACCCACGTT	Identify cDCs; antigen presentation	148227
CD24	M1/69	TATATCTTTGCCGCA	DCs	101841
CD103	2E	TTCATTAGCCCGCTG	DCs	121437
CD64	X54-5/7.1	AGCAATTAACGGGAG	macrophage marker	139329
CD83	Michel-19	TCTCAGGCTTCCTAG	mature DCs and activated lymphocytes	121519
M-HTO-1	M1/42; 30-F11	ACCCACCAGTAAGAC	Mouse hashtag 1	155801
M-HTO-2	M1/42; 30-F11	GGTCGAGAGCATTCA	Mouse hashtag 2	155803
M-HTO-3	M1/42; 30-F11	CTTGCCGCATGTCAT	Mouse hashtag 3	155805
M-HTO-4	M1/42; 30-F11	AAAGCATTCTTCACG	Mouse hashtag 4	155807
M-HTO-5	M1/42; 30-F11	CTTTGTCTTTGTGAG	Mouse hashtag 5	155809
M-HTO-6	M1/42; 30-F11	TATGCTGCCACGGTA	Mouse hashtag 6	155811
