# Supplementary material for: Targeting CXCL16 and STAT1 augments immune checkpoint blockade therapy in triple-negative breast cancer
Source: Nat Commun. 2023 Apr 13;14:2109. doi: 10.1038/s41467-023-37727-y (PMC10101955; doi:10.1038/s41467-023-37727-y)
Supplement: Supplementary file 3 — Description of Additional Supplementary Files [file 41467_2023_37727_MOESM3_ESM.pdf]

Description of Additional Supplementary Files

File Name: Supplementary Data 1
Description: Differential gene expression analysis of different Seurat RNA Clusters 0 to 16 (one tab per Cluster). Included is a list of genes, their log fold change and the references used to identify the clusters.

File Name: Supplementary Data 2
Description: ssGSEA pathway enrichment analysis of myeloid (defined by RNA) clusters in Seurat analysis.

File Name: Supplementary Data 3
Description: Differential gene expression analysis of the Subsetted clusters 0 and 4 which showed presence of 8 unique clusters.

File Name: Supplementary Data 4
Description: ssGSEA pathway enrichment analysis of reclustered Clusters 0 and 4.

File Name: Supplementary Data 5
Description: Gene regulatory network analysis (SCENIC analysis) on Clusters 0, 8, 1. Here gene sets with normalized enrichment scores (NES) greater than 4 are shown.

File Name: Supplementary Data 6
Description: Human patient sample information showing the tumor histology and the treatment received by the patient.

File Name: Supplementary Data 7
Description: Gene regulatory analysis showing the Cluster 0 transcription factor, Stat1 targetome genes and their enrichment scores.
