# Supplementary material for: Targeting CXCL16 and STAT1 augments immune checkpoint blockade therapy in triple-negative breast cancer
Source: Nat Commun. 2023 Apr 13;14:2109. doi: 10.1038/s41467-023-37727-y (PMC10101955; doi:10.1038/s41467-023-37727-y)
Supplement: Supplementary file 11 — Reporting Summary [file 41467_2023_37727_MOESM11_ESM.pdf]

nature portfolio

Corresponding author(s): Siyuan Zhang

Last updated by author(s): Feb 25, 2023

Reporting Summary

Nature Portfolio wishes to improve the reproducibility of the work that we publish. This form provides structure for consistency and transparency in reporting. For further information on Nature Portfolio policies, see our Editorial Policies and the Editorial Policy Checklist.

Statistics

For all statistical analyses, confirm that the following items are present in the figure legend, table legend, main text, or Methods section.

n/a	Confirmed	
☐	☒	The exact sample size (*n*) for each experimental group/condition, given as a discrete number and unit of measurement
☐	☒	A statement on whether measurements were taken from distinct samples or whether the same sample was measured repeatedly
☐	☒	The statistical test(s) used AND whether they are one- or two-sided<br>*Only common tests should be described solely by name; describe more complex techniques in the Methods section.*
☒	☐	A description of all covariates tested
☒	☐	A description of any assumptions or corrections, such as tests of normality and adjustment for multiple comparisons
☐	☒	A full description of the statistical parameters including central tendency (e.g. means) or other basic estimates (e.g. regression coefficient) AND variation (e.g. standard deviation) or associated estimates of uncertainty (e.g. confidence intervals)
☐	☒	For null hypothesis testing, the test statistic (e.g. *F*, *t*, *r*) with confidence intervals, effect sizes, degrees of freedom and *P* value noted<br>*Give P values as exact values whenever suitable.*
☒	☐	For Bayesian analysis, information on the choice of priors and Markov chain Monte Carlo settings
☐	☒	For hierarchical and complex designs, identification of the appropriate level for tests and full reporting of outcomes
☐	☒	Estimates of effect sizes (e.g. Cohen's *d*, Pearson's *r*), indicating how they were calculated

Our web collection on statistics for biologists contains articles on many of the points above.

Software and code

Policy information about availability of computer code

Data collection	Illumina local run manager for sequencing data collection. The R code for data analysis is available at: https://github.com/S-Zhang-Lab/MCT-myeloid upon publication.
Data analysis	Cell Ranger 3.1 and 6 (10X genomics) and R software with Seurat V4 package for single cell CITE-seq analysis

For manuscripts utilizing custom algorithms or software that are central to the research but not yet described in published literature, software must be made available to editors and reviewers. We strongly encourage code deposition in a community repository (e.g. GitHub). See the Nature Portfolio guidelines for submitting code & software for further information.

Data

Policy information about availability of data

All manuscripts must include a data availability statement. This statement should provide the following information, where applicable:

- Accession codes, unique identifiers, or web links for publicly available datasets
- A description of any restrictions on data availability
- For clinical datasets or third party data, please ensure that the statement adheres to our policy

All CITE-sequencing Data has been deposited at GEO (accession code GSE158888). Link to the accession code: https://www.ncbi.nlm.nih.gov/geo/query/acc.cgi?acc=GSE158888. This data will become public from March 1, 2023.

Human research participants

Policy information about studies involving human research participants and Sex and Gender in Research.

Reporting on sex and gender	Not applicable
Population characteristics	Not applicable
Recruitment	Not applicable
Ethics oversight	Not applicable

Note that full information on the approval of the study protocol must also be provided in the manuscript.

Field-specific reporting

Please select the one below that is the best fit for your research. If you are not sure, read the appropriate sections before making your selection.

☒ Life sciences ☐ Behavioural & social sciences ☐ Ecological, evolutionary & environmental sciences

For a reference copy of the document with all sections, see nature.com/documents/nr-reporting-summary-flat.pdf

Life sciences study design

All studies must disclose on these points even when the disclosure is negative.

Sample size	Sample size was determined by pilot experiments and power analysis.
Data exclusions	No data exclusion.
Replication	Minimally 3 biological replicates are included for each experimental design and all the attempts were successful.
Randomization	Mice were randomly assigned to experimental groups.
Blinding	Blinding was performed for all the experiments while allocating during data collection and analysis. In data analysis (single cell sequencing and tumor volume calculation) blinding is not relevant.

Reporting for specific materials, systems and methods

We require information from authors about some types of materials, experimental systems and methods used in many studies. Here, indicate whether each material, system or method listed is relevant to your study. If you are not sure if a list item applies to your research, read the appropriate section before selecting a response.

Materials & experimental systems

n/a	Involved in the study	
☐	☒	Antibodies
☐	☒	Eukaryotic cell lines
☒	☐	Palaeontology and archaeology
☐	☒	Animals and other organisms
☐	☒	Clinical data
☒	☐	Dual use research of concern

Methods

n/a	Involved in the study	
☒	☐	ChIP-seq
☐	☒	Flow cytometry
☒	☐	MRI-based neuroimaging

Antibodies

Antibodies used	CITE-Seq antibodies used are below (antibody name, clone, oligonucleotide sequence, motivation, catalogue number): CCR2/CD192 (SA203G11, AGTGCGATCTGCAAC, Peripheral macrophage, 150625), CD117/c-kit (2B8, TGCATGTCATCGGTG, Innate lymphoid cells (ILCs), 105843), CD11b (M1/70, TGAAGGCTCATTTGT, General myeloid marker, 101265), CD11c (N418, GTTATGGACGCTTGC, DCs, 117355), CD172a/SIRP (P84, GATTCCCTTGTAGCA, Don't eat me signal (SIRP), 144033), CD25 (PC61, ACCATGAGACACAGT, T Cell activation, 102055), CD3 (17A2, GTATGTCCGCTCGAT, General T cell, 100251), CD4 (RM4-5, AACAAGACCCTTGAG, T helper or T reg, 100569), CD44 (IM7, TGGCTTCAGGTCCTA, DC activation marker, 103045), CD45 (30-F11, TGGCTATGGAGCAGA, General immune cell marker, 103159), CD45R/B220 (RA3-6B2, CCTACACCTCATAAT, B cells,pDCs

103263), CD86 (GL-1 , CTGGATTTGTGTATC, Costimulation, 105047), CD8a (53-6.7, TACCCGTAATAGCGT, Cytotoxic T cells, pDCs, 100773), CD90.1 (OX-7, AGTATGGGATGCAAT, DC activation, 202547), Cx3cr1 (SA011F11, CACTCTCAGTCCTAT, DC chemokine receptor, 149041), F4/80 (BM8, TTAACTTCAGCCCGT, General macrophage, 123153), I-A/I-E (M5/114.15.2, GGTCACCAGTATGAT, MHC II, high in cDCs, 107653), Ly6C (HK1.4, AAGTCGTGAGGCATG, Monocyte subset, 128047), Ly6G (1A8, ACATTGACGCAACTA, Neutrophils, 127655), NK1.1 (PK136, GTAACATTACTCGTC, NK cells, 108755), PD-1 (RMP1-30, GAAAGTCAAAGCACT, Co-inhibitory signaling, 109123), PD-L1 (MIH6, TCGATTCCACCAACT, Co-inhibitory signaling, 153604), CD169/Siglec-1 (3D6.112, ATTGACGACAGTCAT, Macrophage cell adhesion,self-tolerance, 142425), Siglec-H (551, CCGCACCTACATTAG, pDCs, 129615), XCR1 (Zet, TCCATTACCCACGTT, Identify cDCs; antigen presentation, 148227), CD24 (M1/69, TATATCTTTGCCGCA, DCs, 101841), CD103 (2E, TTCATTAGCCCGCTG, DCs, 121437), CD64 (X54-5/7.1, AGCAATTAACGGGAG, macrophage marker, 139329), CD83 (Michel-19, TCTCAGGCTTCCTAG, mature DCs and activated lymphocytes, 121519), M-HTO-1 (M1/42; 30-F11, ACCCACCAGTAAGAC, Mouse hashtag 1, 155801), M-HTO-2 (M1/42; 30-F11, GGTCGAGAGCATTCA, Mouse hashtag 2, 155803), M-HTO-3 (M1/42; 30-F11, CTTGCCGCATGTCAT, Mouse hashtag 3, 155805), M-HTO-4 (M1/42; 30-F11, AAAGCATTCTTCACG, Mouse hashtag 4, 155807), M-HTO-5 (M1/42; 30-F11, CTTTGTCTTTGTGAG, Mouse hashtag 5, 155809), M-HTO-6 (M1/42; 30-F11, TATGCTGCCACGGTA, Mouse hashtag 6, 155811).

CyTOF antibodies bought from Fludigm (their conjugates, catalog number, clones): CD45 (089Y-3089005B, 30-F11), CD45R (144Nd, 3144011B, RA3-6B2), Ly-6G (141Pr, 3141008B, 1A8), Ly-6C (162Dy, 3162014B, HK1.4), CD11b (148Nd, 3148003B, M1/70), NK1.1 (165Ho, 3165018B, PK136), I-A/I-E (209Bi, 3209006B, M5/114.15.2), CD11c (967 142Nd, 3142003B, N418), CD3e (152Sm , 3152004B, 145-2C11), CD4 (145Nd, 3145002B, RM4-5), CD25 (151Eu, 3151007B, 3C7), CD44 (150Nd, 3150018B, IM7), CTLA-4 (154Sm, 3154008B, UC10-4B9), CD8a (168Er, 3168003B, 53-6.7), CD86 (172Yb, 3172016B, GL1), CX3CR1 (164Dy, 3164023B, SA011F11), PD-1 (159Tb, 3159024B, 29F.1A12), PD-L1 (153Eu, 3153016B, 10F.9G2), CD117- ckit (166Er, 3166004B, 2B8).

Immunihistochemistry staining: The primary antibodies used were Ki-67 (dilution 1:800, clone D3B5, Cell Signaling Technology, CST 12202S) and anti-CD3 (dilution 1:500, Abcam, ab 16669, Rabbit mAb, SP7).
Immunofluorescence staining: immunanti-Iba1 (dilution 1:2000, Abcam ab5076, Goat polyclonal immunogen to NP_001614 and NP_116573), Phosphorylated STAT1 Stat1 - Ser 727(dilution 1:200, BioLegend 686405, A15158B), anti-CD3 (dilution 1:500, Abcam,ab 16669, Rabbit mAb, SP7), anti-CD8 (dilution 1:250, CST 98941S, Rabbit mAb, D4W2Z), anti-PD-L1 (dilution 1:500, CST 64988S, Rabbit mAb, D5V3B), and anti-CXCL16 (dilution 1:500, R&D Systems MAB503-100, 142417).
Cell Proliferation tracing by CFSE stain (ThermoFisher, Catalog#C34554, 1:1000 of stock solution).
Flow Cytometry: The antibodies used for analysis on FC500 were: Zombie Aqua viability dye (BioLegend, 423101, 1:200), PE/APC anti-mouse CD45 antibody (BioLegend, 103105/103112, 30-F11, 1:100), Alexa Fluor 488 anti-mouse CD11c antibody (BioLegend, 149021, N418, 1:50), PE/Cy7 anti-mouse CD11c (BioLegend, 117318, N418, 1:50), APC/Cy7 CD11b antibody (BioLegend, 101225, M1/70, 1:100), APC anti-mouse CD103 (BioLegend, 121413, 2E7, 1:20), APC/Cy7 CD8 anti-mouse antibody (BioLegend, 100713, 53-6.7, 1:100), PE anti-mouse PD-L1 (BioLegend, 124308, 10F.9G2, 1:100), Alexa Fluor 488 pStat1 (Ser 727) (Biolegend, 686410, A15158B, 1:50), PE anti-CD44 anti-mouse antibody (BioLegend, 10323, IM7, 1:100). For analysis on Cytek Flow cytometer, Myeloid cell specific markers used were: BV421 anti-mouse CD86 (BioLegend,105031, GL-1, 1:100), BV570 anti-mouse CD11c (BioLegend,117331, N418, 1:50) or 11b (BioLegend,101233, M1/70, 1:100), BV605 anti-mouse CD11b (BioLegend,101257, M1/70, 1:100) or 11c (BioLegend,117333, N418, 1:50), BV711 anti-mouse PD-L1(BioLegend,124319, 10F.9G2, 1:100), FITC anti-mouse Ccr1 (BioLegend,152505, S15040E, 1:50), PE anti-mouse CXCL16 (BD, 566740, 12-81, 1:20), PE-Dazzle/594 I-A-I-E (BioLegend,107647, M5/114.15.2, 1:100), PerCP-Cy5.5 anit-mouse pStat1(p727) (BioLegend,686415, A15158B, 1:100), PE-Cy7 CD45 (BioLegend,157206, S18009F, 1:100); Lineage specific markers used were Pacific Blue anti-mouse Lin (BioLegend,133305, 17A2 ; RB6-8C5 ; RA3-6B2 ; Ter-119 ; M1/70, 1:100) and BV510 anti-mouse c-KIT (BioLegend,105839, 2B8, 1:100); T cell specific markers used were BV 421 PD-1 (BioLegend,135217, 29F.1A12, 1:100), BV510 CD69 (BioLegend,104531, H1.2F3, 1:100), FITC anti-mouse CD45 (BioLegend,103122, 30-F11, 1:100), PE/Cy5 CD4 (BioLegend,100410, GK1.5, 1:100), PE-Dazzle 594 CD8a (BioLegend,100762, 53-6.7, 1:100), PerCP-Cy5.5 CXCR6 (BioLegend,151120, SA051D1, 1:100), PE-Cy7 anti-mouse CD3e (BioLegend,100319, 145-2C11, 1:100), APC/Fire 750 anti-mouse CD3 (BioLegend, 100248, 17A2, 1:100), immune cell marker BV785 CD45.1 (BioLegend,110743, A20, 1:100), and T cell activation marker BV711 IFN-γ (BioLegend, 505835, XMG1.2, 1:50), BV570 CD44 (BioLegend, 103037, IM7, 1:100), BV785 anti-mouse/human KLRG1 (BioLegend, 138429, 2F1/KLRG1, 1:100), APC CD49 (BioLegend, 142606, HMa1, 1:100), Pacific Blue CD45 (BioLegend, 103126, 30-F11, 1:100), PE anti-mouse CD103 (BioLegend, 121406, 2E7, 1:20). For tetramer panel APC/Fire 750 anti-mouse CD3 (BioLegend, 100248, 17A2, 1:100) and BV421 Flex-T™ H-2 K(b) OVA (SIINFEKL) Tetramer (BioLegend, 280051, 1:100) were used.

Validation	All flow cytometry antibodies and CITE-seq antibodies were purchased from BioLegend, ThermoFisher, and BD Biosciences. The validation experiments have been conducted by the manufacturer itself.

Eukaryotic cell lines

Policy information about cell lines and Sex and Gender in Research

Cell line source(s)	E0771 Mouse tumor cell line from CH3 BioSystems
Authentication	CellCheck TM Mouse Plus service provided by IDEXX BioResearch helps check for cell line contamination with cell lines of other species.
Mycoplasma contamination	Tested negative
Commonly misidentified lines (See ICLAC register)	No commonly misidentified cell lines were used in the study.

Animals and other research organisms

Policy information about studies involving animals; ARRIVE guidelines recommended for reporting animal research, and Sex and Gender in Research

Laboratory animals	FVB-MMTV-neu, FVB.C3Tag, C57B6, C57.PyMT, OT-I. All animals were purchased from Jax laboratory. They were housed in the animal facility with 12 hours of light and 12 hours of night cycle and food/water was available all the time.
Wild animals	No wild animals were used in the study.
Reporting on sex	We only used female mice in the study
Field-collected samples	No field-collected samples were used in the study.
Ethics oversight	All the mouse experiments were performed as per University of Notre Dame (Protocol # 18-05-4687) and UT Southwestern's (Protocol #2022-103340) Institutional Animal Care and Use Committee (IACUC) approved protocols. The study with TNBC clinical specimens in this publication was approved by the UT Southwestern Institutional Review Board (IRB# STU102010-051).

Note that full information on the approval of the study protocol must also be provided in the manuscript.

Clinical data

Policy information about clinical studies

All manuscripts should comply with the ICMJE guidelines for publication of clinical research and a completed CONSORT checklist must be included with all submissions.

Clinical trial registration	Not applicable
Study protocol	Not applicable
Data collection	Not applicable
Outcomes	Not applicable

Flow Cytometry

Plots

Confirm that:

☒ The axis labels state the marker and fluorochrome used (e.g. CD4-FITC).

☒ The axis scales are clearly visible. Include numbers along axes only for bottom left plot of group (a 'group' is an analysis of identical markers).

☒ All plots are contour plots with outliers or pseudocolor plots.

☒ A numerical value for number of cells or percentage (with statistics) is provided.

Methodology

Sample preparation	Tissue digestion followed by single cell isolation or immune cell enrichment via magnets based methods.
Instrument	Cytek Northern Light
Software	Flowjo
Cell population abundance	>10,000 cells were analyzed in each experiment.
Gating strategy	All the events were gated on FSC/SSC for cells/lymphocytes, then gated on FSC-H/FSC-A for singlets, Zombie Aqua low for live cells, then on CD45 high (immune cells) followed by either CD3 (T cells) or CD11b (myeloid) for subsets. They have been further gated into their subsets into CD4/CD8 or CD11c-IAIE/CXCL16. Detailed gating strategies have been included in the supplementary figure. For proliferated T cells upon CD4/CD8 gating, CFSE peaks were used gate them into those proliferated either once, twice, or thrice.

☒ Tick this box to confirm that a figure exemplifying the gating strategy is provided in the Supplementary Information.
